# Soluble RAGE as a severity marker in community acquired pneumonia associated sepsis

**DOI:** 10.1186/1471-2334-12-15

**Published:** 2012-01-20

**Authors:** Rodrigo M Narvaez-Rivera, Adrian Rendon, Mario C Salinas-Carmona, Adrian G Rosas-Taraco

**Affiliations:** 1 Universidad Autonoma de Nuevo Leon, UANL, School of Medicine and University Hospital, Department of Internal Medicine. Monterrey Nuevo Leon. Mexico; 2 Universidad Autonoma de Nuevo Leon, UANL, School of Medicine and University Hospital, CIPTIR (Centro de Investigacion, Prevencion y Tratamiento de Infecciones Respiratorias). Monterrey, Nuevo Leon, Mexico; 3 Universidad Autonoma de Nuevo Leon, UANL, School of Medicine and University Hospital, Department of Immunology. Monterrey Nuevo Leon. Mexico

**Keywords:** SOFA score, Soluble RAGE, Severity markers, Community-acquired pneumonia, Survival

## Abstract

**Background:**

Community-acquired pneumonia (CAP) is considered the most important cause of death from infectious disease in developed countries. Severity assessment scores partially address the difficulties in identifying high-risk patients. A lack of specific and valid pathophysiologic severity markers affect early and effective sepsis therapy. HMGB-1, sRAGE and RAGE have been involved in sepsis and their potential as severity markers has been proposed. The aim of this study was to evaluate HMGB-1, RAGE and sRAGE levels in patients with CAP-associated sepsis and determine their possible association with clinical outcome.

**Method:**

We evaluated 33 patients with CAP-associated sepsis admitted to the emergency room and followed in the medical wards. Severity assessment scores (CURB-65, PSI, APACHE II, SOFA) and serologic markers (HMGB-1, RAGE, sRAGE) were evaluated on admission.

**Results:**

Thirty patients with a diagnosis of CAP-associated sepsis were enrolled in the study within 24 hours after admission. Fourteen (46.6%) had pandemic (H1N1) influenza A virus, 2 (6.6%) had seasonal influenza A and 14 other diagnoses. Of the patients in the study group, 16 (53.3%) had a fatal outcome. ARDS was observed in 17 (56.6%) and a total of 22 patients had severe sepsis on admission (73%). The SOFA score showed the greatest difference between surviving and non-surviving groups (*P *= .003) with similar results in ARDS patients (*P *= .005). sRAGE levels tended to be higher in non-surviving (*P *= .058) and ARDS patients (*P *= .058). Logistic regression modeling demonstrated that SOFA (*P *= .013) and sRAGE (*P *= .05) were the only variables that modified the probability of a fatal outcome.

**Conclusion:**

The association of elevated sRAGE with a fatal outcome suggests that it may have an independent causal effect in CAP. SOFA scores were the only clinical factor with the ability to identify surviving and ARDS patients.

## Background

Community-acquired pneumonia (CAP) is considered the leading cause of death from infectious disease in developed countries [1]. In Mexico, the annual estimated incidence is100 to 230 cases per 100, 000 inhabitants, causing an alarming impact on public health since 25% of these cases require hospitalization [2]. Severity assessment scores help identify high-risk patients that need hospital therapy; however, the lack of specific and valid pathophysiologic severity markers affects early effective interventions. The recent H1N1 influenza pandemic (p2009A H1N1 or S-OIV) was associated with an increase in cases of CAP that required hospitalization and continues to be a national public health threat [3-6]. Although the mortality rate was only 1.8%, 31% of patients with severe disease were admitted to an intensive care unit, and 14%-46% died [7-10]. The first 18 cases, seen from March 24 to April 24, 2009 were reported at the National Institute of Respiratory Diseases in Mexico City. More than half of the patients were between 13 and 47 years of age. Twelve patients required mechanical ventilation and seven died (38%) [3]. Increased mortality was associated with systemic manifestations and complications of CAP with sepsis being the most common and challenging.

Physicians may underestimate the severity of CAP, which can lead to insufficiently aggressive interventions inpatients with a high risk of complications [11,12]. Scoring systems have been used to calculate the probability of morbidity or mortality. The most studied scoring system, the Pneumonia Severity Index (PSI), is a 20-point score that classifies patients into five risk categories based on their percentage of risk of death within 30 days. This score was useful in patients with a low risk of death (0.1%-0.7%) and was recommended for outpatient therapy [13]. However, PSI is limited by its number of variables, making it complex for the emergency room setting [14].

The British Thoracic Society subsequently designed a simpler prediction tool, the confusion, urea, respiration, and blood pressure (CURB) score, also based on the risk of 30 day mortality [12]. In 2003, Lim and colleagues added age ≥65 years as a risk factor to create CURB-65 [15]. CURB-65 is significantly easier to use than PSI since it has only five variables with a single point awarded for each. CURB-65 is recommended together with PSI.

Other severity assessments, such as the Acute Physiology and Chronic Health Evaluation II (APACHE II), are commonly used in intensive care units to determine a patient's outcome. The Sequential Organ Failure Assessment score (SOFA) on admission has also been used with results similar to APACHE II. The combination of these may improve sensitivity [16].

Current severity assessment scores only partially overcome the difficulties in identifying patients with severe disease, providing objective classifications of patients into high-risk categories [17]. Thus, there is increasing interest in improving diagnostic accuracy by measuring inflammatory mediators that participate in sepsis. In 1999, Wang et al. reported that high-mobility group box 1 (HMGB1) was detectable in plasma of mice exposed to a lipopolysaccharide. Removal of circulating HMGB1 with a specific antibody improved survival. HMGB1 has delayed kinetics and remains in circulation longer than the initial studied immunologic mediators. HMGB-1 induces the release of proinflammatory and procoagulant factors and when injected into mice, leads to the development of clinical features of sepsis and multiorgan dysfunction [18].

Angus and colleagues studied serum HMGB1 levels in a subgroup of 122 patients with CAP and observed elevated levels more than a week after presentation with high circulating levels associated with greater mortality [19]. These data differ from Sunden-Cullberg et al., who found lower HMGB1 serum levels in non-survivors of severe sepsis [20]. There have also been studies evaluating the role of HMGB-1's receptor, the receptor for advanced glycation end products (RAGE) [21]. Experimental studies demonstrate that RAGE-dependent activation of nuclear factor-kappa B (NF-κB) plays a central role in modulating mortality after cecal ligation and puncture [22]. RAGE possesses a secretory isoform known as soluble RAGE (sRAGE), which maintains the extracellular ligand-binding domain but lacks the cytosolic and transmembrane domains. sRAGE has the same ligand binding specificity and competes with cell-bound RAGE, serving as a decoy that abolishes cell activation. In sepsis models, the administration of exogenous sRAGE slightly improved survival [22]. Evidence suggests that human endogenous sRAGE is generated by alternative splicing of RAGE mRNA, or alternatively, by proteolytic cleavage from membranous RAGE [23]. This former mechanism was considered to be a cell regulating mechanism, permitting restoration of homeostasis and survival.

Since there is very little knowledge of the role of HMGB-1/RAGE in the clinical setting of CAP-associated sepsis, we decided to perform a pilot study to investigate of HMGB-1, RAGE and sRAGE levels in septic patients with CAP and identify if there is a correlation with severity assessment scores.

## Methods

### Study groups

This observational clinical study included patients evaluated at the UANL University Hospital in Monterrey, Mexico. The Bioethics Committee of the School of Medicine of the Universidad Autonoma de Nuevo Leon previously approved this project and written informed consent was obtained from the patient or a legal representative. Thirty-three consecutive patients, from July 2009 through August 2010, were enrolled in the study within the first 24 hours of their arrival to the emergency room with sepsis secondary to CAP. They were followed-up either in the general ward or in the intensive care unit. Patients were classified according to the Sepsis Consensus Conference of 1992 [24] and the Infectious Diseases Society of America. Clinical data, diagnosis, treatment modalities, and blood samples were collected.

The severity of CAP was estimated using the following scores: CURB-65, PSI, APACHE II, and SOFA. To be enrolled, subjects had to be ≥ 18 yrs of age and have both a clinical diagnosis of pneumonia and a new pulmonary infiltrate on chest X-ray. Patients with hospital-acquired pneumonia, an episode of pneumonia in the last 30 days, pulmonary tuberculosis, pregnancy, palliative care, cancer, human immunodeficiency virus infection, chronic steroid use, acute or chronic viral liver disease, and chronic renal disorders were excluded from the study.

At enrollment, blood samples were taken, and RAGE receptor was immediately detected by flow cytometry, determining its mean fluorescence intensity. Subsequently HMGB-1 and sRAGE antigen were determined in plasma by enzyme-linked immunosorbent assay (ELISA). At the same time, CURB-65, PSI, APACHE II score, and SOFA score were documented. During the patient's hospital stay we evaluated the presence of acute respiratory distress syndrome (ARDS). Also, a follow-up at 28 days was performed to distinguish between survivors and non-survivors. After enrollment of patients, data was blinded to avoid potential bias.

### HMGB1 and soluble RAGE assay

Blood samples were obtained from each patient and sera were recovered to test HMGB1 and soluble RAGE levels. The HMGB1 ELISA kit (IBL International, Germany) and the soluble RAGE ELISA kit (R&D system, Minneapolis, Mn) were used according to the manufacturer's recommendations.

### Membrane RAGE assay

A sample of whole blood, anticoagulated with EDTA, from each patient was used for flow cytometry analysis. One hundred microliters of whole blood was incubated with a rabbit anti-human RAGE antibody (Chemicon, Billerica, MA) for 15 min at room temperature. A Goat anti-Rabbit IgG FITC conjugate (Chemicon) was used for flow cytometry detection. Samples were incubated for 15 min at room temperature in darkness. Lysis solution was then added to eliminate erythrocytes and two washes with PBS (0.1 M, pH 7.2) were done centrifuging at 220-240 × g for10 min in each time. Leukocytes were recovered by centrifugation in the same condition and the samples were resuspended in 1 ml of FACS flow (BD Biosciences, Pharmingen) for cytofluorometric analysis (FACS SortCalibur, BD, San Jose, CA). Then 10, 000 cells, in which mean fluorescence intensity (MFI) was obtained and nonspecific fluorescence was deleted, were analyzed.

### Statistical analysis

All statistical analyses were performed in SPSS (SPSS, version 13.0), assuming a statistical significance of *P ≤ *.05. The general descriptive characteristics are presented as means, standard deviations, medians, and percentages. We compared the severity assessment scores and HMGB-1, RAGE and sRAGE levels in surviving and non-surviving patients at 28 days, and between ARDS and non-ARDS patients using a statistical inferential analysis with the U Mann-Whitney nonparametric test. Using the normality tests Kolmogorov-Smirnov with the Lilliefors correction and Shapiro-Wilk, we determined if the obtained values came from a normally distributed population. We present data as plots of admission day medians.

We used multivariate logistic regression and Cox regression models with a backward technique to select variables that predicted a fatal outcome, including clinical severity and the inflammatory markers studied, such as age, gender, CURB-65, SOFA score, APACHE II, pneumonia severity index, HMGB-1, sRAGE and RAGE. Correlation between clinical severity scores and immunologic markers at admission, and between the markers, was detected using Spearman's correlation coefficient.

Acute organ dysfunction was defined as a new Sequential Organ Failure Assessment score [25] ≥3 in any of six organ systems, following the European Society of Intensive Care Medicine sepsis occurrence in the acutely ill patient study criteria [26]. We also added patients that met the following alternate definition to the analyses: an increase of 1 Sequential Organ Failure Assessment point in any two organ systems, 2 points in one system, or an absolute score of ≥3 in the respiratory system, similar to criteria used in several large trials of antisepsis agents [27-29].

## Results

### Study population

Thirty-three patients with confirmed CAP were included in the study; three were excluded (one was pregnant and two because of problems with their blood sample). Of the remaining 30 patients, 14 (46.6%) had pandemic (H1N1) 2009 influenza virus confirmed by PCR and 2 patients (6.9%) had seasonal influenza A. No etiologic agent was found in the other 14 patients. Twenty-two patients (73.3%) had severe sepsis or septic shock detected at admission; of these, 17 developed acute respiratory distress syndrome (ARDS). The mortality rate of the study group was a total of 16 patients (53.3%) at the end of the 28 days. There were eight who never developed severe sepsis and survived to hospital discharge, six who developed severe sepsis and survived to discharge, and 16 who developed severe sepsis and died in the hospital.

**Figure 1:**
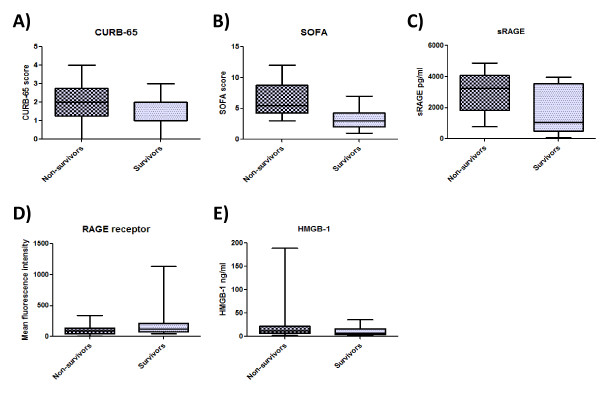
**Analysis of CURB-65 score, SOFA score, soluble RAGE, membrane RAGE and HMGB-1 levels in survival and non-survival patients**. A) CURB-65 and B) SOFA scores were obtained using international protocols. C) Serological soluble RAGE and D) HMGB-1 levels were measured by ELISA. E) Membrane RAGE levels were analyzed by flow cytometry. Data represent the median and were analyzed using Mann Whitney U test.

**Figure 2:**
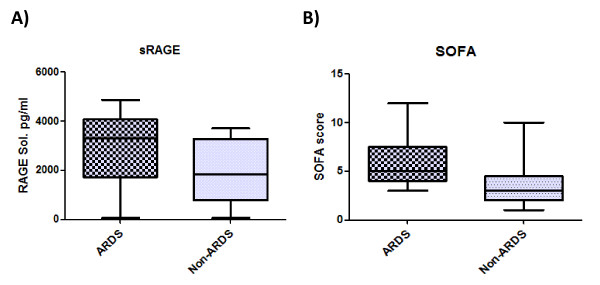
**Analysis of soluble RAGE and SOFA score in ARDS and non-ARDS patients**. Data represent the median and were analyzed using Mann Whitney U test.

There were no significant differences between survival and non-survival patients with respect to age, gender, ethnicity, microbiological etiology, initial CURB-65, initial PSI class, initial APACHE II score, or emergency room length of stay (*P *value range, .07-.99) nor between ARDS and non-ARDS patients with respect to gender, ethnicity, microbiological etiology, initial CURB-65, initial PSI class, initial APACHE II score, or emergency room length of stay (*P *value range, .36-.77) (Figure 1A). Group characteristics are provided in Table 1. Compared with those who did not survive, those who survived had lower SOFA scores (5.5, CI: 4.9-7.7 *versus *3, CI: 2.3-4.2) (Figure 1B). Compared with patients that did not develop ARDS, those with ARDS had higher SOFA scores (3, CI: 2.1-4.9 *versus *5, CI: 4.6-7.1) and were younger (Figure 2B and Table 1).

**Table 1:** Characteristics of the patients in comparative groups: surviving vs non-surviving, ARDS vs non-ARDS

Characteristics	ARDS (n = 17)	Non-ARDS (n = 13)	*P *value	Non-Surviving (n = 16)	Surviving (n = 14)	*P *value
Age, mean (SD)	35.8 (11)	54 (20)		38 (14)	50 (21)	

Male sex^a^	9 (53)	4 (31)		10 (62.5)	3 (21)	

CURB-65	2 (1.45-2.20)	1 (0.89-2.34)	.48	2 (1.49-2.51)	1 (0.94-1.92)	.076

PSI class	3 (2.72-3.98)	2 (2.15-3.85)	.45	4 (2.72-4.28)	2.5 (2.26-3.45)	.224

APACHE II score	11 (10.18-15)	12 (7.54-13.39)	.36	11.5 (9.66 - 15.47)	11.5 (8.42-12.87)	.400

**SOFA score**	**5** **(4.69-7.19)**	**3** **(2.11-4.97)**	**.003**	**5.5** **(4.91-7.71)**	**3** **(2.37-4.20)**	**.001**

Mortality^a^	13 (76)	3 (23)		--------------	--------------	

Renal failure^a^	2 (11)	0 (0)		2 (12)	0 (0)	

Shock^a^	4 (23)	2 (15)		6 (37)	0 (0)	

Respiratory failure^a^	------------	-------------		13 (81)	4 (28.5)	

Microbiological etiology, n (%)						

H1N1 S-OIV 2009^a^	10 (59)	4 (31)		8 (50)	6 (43)	

Seasonal Influenza^a^	2 (12)	0 (0)		2 (12.5)	0 (0)	

Others^a^	5 (29)	9 (69)		6 (37.5)	8 (57)	

APACHE, Acute Physiology and Chronic Health Evaluation; SOFA, Sepsis-Related Organ Failure Assessment; S-OIV, swine origin influenza virus

*^a^*Defined according to the 1992 American College of Chest Physicians/Society of Critical Care Medicine Consensus Conference guidelines^(16)^. Data are mean (SD), median (CI), or n (%) when appropriate

There were no statistically different RAGE, sRAGE and HMGB-1 levels found during early CAP-associated sepsis in ARDS or non-surviving patients (Figure 1C, Figure 1E, Figure 2A and Table 2). No difference was found between influenza A H1N1 infected patients and the rest of the study group (2767 ± 1655 vs 2174 ± 1344, *P *= .327). We did not find a correlation between immunological molecules and severity assessment scores using Spearman's correlation coefficient (*P *value range = .16-.99). Finally, none of the studied severity assessment scores correlated with each other (*P *value range = .18-.79).

**Table 2:** Immunologic marker levels in comparative groups: surviving vs. non-surviving, ARDS vs. non-ARDS

	Non-Surviving Patients [total n = 16] (CI)	Surviving Patients [total n = 14] (CI)	*P *value	Non-ARDS Patients [total n = 13] (CI)	ARDS Patients [total n = 17] (CI)	*P *value
HMGB-1 (ng/mL)^a^	11.76 [13] (-5.65-60)	6.78 [12] (4.5-17.66)	.44	10.11 [11] (4.64-18.97)	11.11 [14] (-4.83-55.51)	.64

RAGE (MFI)^a^	88.88 [15] (55.9-150.8)	116.7 [14] (51.9-456.2)	.21	98.01 [12] (6.68-492.31)	90.63 [17] (75.55-173.27)	.94

sRAGE (pg/mL)^a^	3236 [15] (2312-3673)	1037 [13] (912.8-2769)	.058	1829.75 [13] (1079-2629)	3296 [15] (2168-3793)	.058

HMGB-1, high mobility group box -1, RAGE, receptor for advanced glycation end products; sRAGE, soluble RAGE; MFI, mean fluorescence intensity

[n] Expresses the numeral of results analyzed, from the total number

^a^Data is expressed as median (CI)

### Logistic regression model

Using a logistic regression model involving age, gender, APACHE II, SOFA, HMGB-1, sRAGE and RAGE, we found that the only variables that modified the probability of the patient having a fatal outcome were SOFA (*P *= .013) with a relative risk of surviving of .347 (CI: .151-.797); and sRAGE (*P *= .05) with a relative risk of surviving of .998 (CI: .998-1) (Table 3).

**Table 3:** Logistic Regression Model analysis

	**Sig**.	Exp(B)	95% C.I. Exp(B)	
**sRAGE**	**.050**	.999	.998	1.000

**SOFA score**	**.013**	.347	.151	.797

Constant	.010	2721.943		

Variables included in equation: RAGE, sRAGE, HMGB-1, SOFA score, APACHE II score, gender, age

### Cox regression model

According to multivariate Cox regression analysis we found that a high SOFA score was an independent predictor of non-survival (hazard ratio 1.53, CI: 1.2-1.97, *P *= .001) (Table 4).

**Table 4:** Cox Regression Model analysis

	**Sig**.	Exp(B)	95% C.I. Exp(B)	
**sRAGE**	**.050**	.999	.998	1.000

**SOFA score**	**.013**	.347	.151	.797

Variables included in equation: RAGE, sRAGE, HMGB-1, SOFA score, APACHE II score, gender, age

## Discussion

We found that SOFA scores and the measurement of sRAGE levels in patients with CAP-associated sepsis helped predict survival. To date, this is the first study that analyses the levels of both of these molecules (the "HMGB-1" ligand and the "RAGE" receptor) in the inflammatory cascade of patients with CAP-associated sepsis. In Mexico, as in developed countries, CAP continues to be an important cause of death from infectious disease [1] with an elevated cost to public health. This was particularly evident with the H1N1 (2009, S-OIV) influenza virus pandemic [8]. Overall mortality is about 50% in patients with CAP that develop septic shock [25]. Although there has been intense research on the pathophysiology of CAP and its severe forms, such as ARDS, only slight improvements in new and effective treatment strategies have occurred.

Despite the identification of several recent molecules in patients with infection, such as the receptor expressed on myeloid cells-1 (TREM-1), these lack specificity in sepsis pathophysiology [26-28]. Discovery of markers may add additional information, increasing the validity of clinical estimates and permitting early, aggressive, and effective sepsis therapy. This justifies every effort to further explore the paradigm of biomarkers in the area of pulmonary infections [29]. We still lack efficient tools to identify patients with CAP who are likely to develop severe complications. Current clinical severity scores partially limit these difficulties, but are far from perfect. In our study, CURB-65, APACHE II and PSI demonstrated no difference between groups (fatal outcome and ARDS). Recently published studies have found that CURB-65 dose not reliably distinguish patients with pandemic influenza CAP who will have good or poor outcomes [30,31]. In the case of PSI, this could represent its higher ability to detect mild cases; although, this could be explained by the small number of patients in our study. In contrast, we noticed that SOFA scores, although not specific for CAP, were significantly higher in non-surviving or ARDS patients. Thus, in spite of the wide variety of etiologies, this last organ dysfunction score seems to be useful in patients with CAP.

It is well known that the recognition receptor "RAGE" and HMGB-1 play a central role in the innate immune system with an impact on its perpetuation and amplification [22]. RAGE stimulation results in sustained NF-κB activation, which may be a predictor of severity in sepsis [32]. Conditions that induce NF-κB also increase RAGE expression, which in turn produces sustained inflammation; this is seen in CAP, where RAGE ligands are abundantly present. Angus et al. found that CAP patients had higher HMGB-1 concentrations, and this correlated with mortality [19]. Gaini et al. also found higher levels of HMGB-1 in CAP [33]. Studies of severe influenza CAP demonstrated an association between excessive release of cytokines and increased mortality [34,35]. However, Alleva et al. found in a murine model of severe influenza that HMGB-1 concentrations were not increased in plasma at the time of peak mortality, and peak levels of HMGB1 did not occur until relatively late in infection [36].

Recently, Bopp et al. demonstrated that sRAGE concentrations in sepsis patients were higher in non-survivors when compared with survivors. They concluded that larger clinical trials should study the potential role of sRAGE as a new sepsis marker [37]. However, sRAGE has been used in animal models to block HMGB-1's binding to the RAGE receptor, leading to increased survival. This data indicates that HMGB-1 and RAGE participate in sepsis, including sepsis patients with CAP.

After developing multivariate regression models using backward selection techniques, we found that sRAGE and SOFA predicted survival; although the statistical significance was greater for SOFA, a limitation of our study is the small number of patients. One explanation for the elevated sRAGE levels could be an increased gene expression of RAGE in patients with sepsis [22,38]. Since we know that RAGE participates in tissue damage [39], it could represent a marker for cellular damage in sepsis.

As mentioned previously, there were elevated concentrations of sRAGE on admission in those with a fatal outcome, but without statistical significance. The same was observed in those patients who developed ARDS. On the other hand, receptor RAGE and HMGB-1 demonstrated lower differences between groups. Larger studies will be necessary to investigate the role of these potential sepsis markers.

The elevated levels of sRAGE found in our study, as in others, might represent the septic status of the patients as splice-variants of RAGE or shed variants of cell surface RAGE. In contrast to animal studies where a protective effect of sRAGE was seen, we found that sRAGE levels were higher in patients with more inflammation and in non-survivors. This finding could be related to shed variants of cell surface RAGE but this aspect was not one of our objectives. The ELISA we used did not differentiate between splicing variants and the shed variants of RAGE.

To the best of our knowledge, this is the second study that finds higher sRAGE levels in plasma of sepsis non-survivors compared with survivors [37]. This has discrepancies with mouse model studies of sepsis after CLP [22]. This could be in part explained by different kinds of sepsis, different etiologic agents, and what was difficult to determine in our study, the time of measurement after the immunologic process started.

We do not know if sRAGE concentrations were enough to bind HMGB-1, after they had scavenged AGEs and other RAGE ligands. Moreover, the higher concentrations found in sicker patients could represent sRAGE modified structurally and functionally during sepsis, diminishing its binding and neutralizing capacity.

## Conclusions

Plasma sRAGE levels are elevated in CAP patients. sRAGE performed as an independent factor affecting the probability of a fatal outcome. Interestingly, the SOFA score demonstrated greater accuracy with the ability to differentiate between surviving/non-surviving and ARDS/non-ARDS groups.

## Abbreviations

APACHE II score: Acute Physiology and Chronic Health Evaluation II; ARDS: Acute respiratory distress syndrome; CAP: Community-acquired pneumonia; CURB-65: Confusion, urea, respiration, blood pressure and age ≥65 years; EDTA: Ethylenediaminetetraacetic acid: ELISA: Enzyme-Linked ImmunoSorbent Assay; FACS flow: Fluorescence-Activated Cell Sorting, *flow *cytometry; FITC: Fluorescein isothiocyanate; HMGB-1: High-mobility group box 1; MFI: Mean fluorescence intensity; NF-κB: Nuclear factor-kappa B; PSI: Pneumonia Severity Index; RAGE: Receptor for advanced glycation end products; sRAGE: Soluble RAGE.

## Competing interests

The authors declare that they have no competing interests.

## Authors' contributions

RN, conception of the study, developed the analytic plan, participated in acquisition and interpretation of data, performed statistical analysis, helped draft the manuscript. AR and MCS contributed to the conception and the design. AGR carried out the immunoassays, conception of the study, its design, and data analysis. All authors helped with interpretation of data, revising it critically for important intellectual content, drafted the manuscript, and read and approve the final manuscript.

## Authors' Information

RN, M.D. last year resident of internal medicine in the University Hospital of the Universidad Autonoma de Nuevo Leon (UANL), in Monterrey, Mexico. AR, M.D. internal medicine, pulmonary and critical care professor; Head of CIPTIR (Centro de Investigación, Prevención y Tratamiento de Infecciones Respiratorias). School of Medicine and University Hospital, UANL. Monterrey, Nuevo Leon, Mexico. MCS, M.D. and PhD in Immunology. Head of Department of Immunology, School of Medicine and University Hospital, UANL. Monterrey, Nuevo Leon. Mexico. AGR, PhD in Immunology. Field: Immune response to infectious diseases. Professor in Immunology and Head of Molecular Immunology Laboratory. Department of Immunology, School of Medicine and University Hospital, UANL. Monterrey, Nuevo Leon. Mexico.

## Pre-publication history

The pre-publication history for this paper can be accessed here:

http://www.biomedcentral.com/1471-2334/12/15/prepub

## References

[B1] Mandell LA Infectious diseases society of America/American thoracic society consensus guidelines on the management of community-acquired pneumonia in adults Clin Infect Dis 2007 44 Suppl 2 S27 72 17278083 10.1086/511159 PMC7107997

[B2] Epidemiologic Vigilance 2003 DGE/SSA in Mexico 51st week

[B3] Perez-Padilla R Pneumonia and respiratory failure from swine-origin influenza a (H1N1) in Mexico N Engl J Med 2009 361 7 680 689 10.1056/NEJMoa0904252 19564631

[B4] Martinez-Hernandez F What happened after the initial global spread of pandemic human influenza virus A (H1N1)? a population genetics approach Virol J 2010 7 196 10.1186/1743-422X-7-196 20727188 PMC2936310

[B5] Pandemic influenza: the new wave Lancet Infect Dis 2009 9 10 583 10.1016/S1473-3099(09)70236-1 19778756

[B6] Dominguez-Cherit G H1N1 influenza pandemic of 2009 compared with other influenza pandemics: epidemiology, diagnosis, management, pulmonary complications, and outcomes Curr Infect Dis Rep 2010 12 3 204 210 10.1007/s11908-010-0097-0 21308531 PMC7101813

[B7] Bautista E Clinical aspects of pandemic 2009 influenza A (H1N1) virus infection N Engl J Med 2010 362 18 1708 1719 20445182 10.1056/NEJMra1000449

[B8] Kumar A Critically ill patients with 2009 influenza A(H1N1) infection in Canada JAMA 2009 302 17 1872 1879 10.1001/jama.2009.1496 19822627

[B9] Webb SA Critical care services and 2009 H1N1 influenza in Australia and New Zealand N Engl J Med 2009 361 20 1925 1934 19815860 10.1056/NEJMoa0908481

[B10] Rello J Intensive care adult patients with severe respiratory failure caused by Influenza A (H1N1)v in Spain Crit Care 2009 13 5 R148 10.1186/cc8044 19747383 PMC2784367

[B11] Tang CM Macfarlane JT Early management of younger adults dying of community acquired pneumonia Respir Med 1993 87 4 289 294 10.1016/0954-6111(93)90025-U 9728229

[B12] Neill AM Community acquired pneumonia: aetiology and usefulness of severity criteria on admission Thorax 1996 51 10 1010 1016 10.1136/thx.51.10.1010 8977602 PMC472650

[B13] Fine MJ A prediction rule to identify low-risk patients with community-acquired pneumonia N Engl J Med 1997 336 4 243 250 10.1056/NEJM199701233360402 8995086

[B14] Lee RW Lindstrom ST A teaching hospital's experience applying the pneumonia severity index and antibiotic guidelines in the management of community-acquired pneumonia Respirology 2007 12 5 754 758 10.1111/j.1440-1843.2007.01121.x 17875067

[B15] Lim WS Defining community acquired pneumonia severity on presentation to hospital: an international derivation and validation study Thorax 2003 58 5 377 382 10.1136/thorax.58.5.377 12728155 PMC1746657

[B16] Ho KM Combining sequential organ failure assessment (SOFA) score with acute physiology and chronic health evaluation (APACHE) II score to predict hospital mortality of critically ill patients Anaesth Intensive Care 2007 35 4 515 521 18020069 10.1177/0310057X0703500409

[B17] Singanayagam A Chalmers JD Hill AT Severity assessment in community-acquired pneumonia: a review QJM 2009 102 6 379 388 10.1093/qjmed/hcp027 19299247

[B18] Wang H HMG-1 as a late mediator of endotoxin lethality in mice Science 1999 285 5425 248 251 10.1126/science.285.5425.248 10398600

[B19] Angus DC Circulating high-mobility group box 1 (HMGB1) concentrations are elevated in both uncomplicated pneumonia and pneumonia with severe sepsis Crit Care Med 2007 35 4 1061 1067 10.1097/01.CCM.0000259534.68873.2A 17334246

[B20] Sunden-Cullberg J Persistent elevation of high mobility group box-1 protein (HMGB1) in patients with severe sepsis and septic shock Crit Care Med 2005 33 3 564 573 10.1097/01.CCM.0000155991.88802.4D 15753748

[B21] Bierhaus A Stern DM Nawroth PP RAGE in inflammation: a new therapeutic target? Curr Opin Investig Drugs 2006 7 11 985 991 17117586

[B22] Liliensiek B Receptor for advanced glycation end products (RAGE) regulates sepsis but not the adaptive immune response J Clin Invest 2004 113 11 1641 1650 15173891 10.1172/JCI18704 PMC419481

[B23] Hudson BI Identification, classification, and expression of RAGE gene splice variants FASEB J 2008 22 5 1572 1580 18089847 10.1096/fj.07-9909com

[B24] Bone RC Sibbald WJ Sprung CL The ACCP-SCCM consensus conference on sepsis and organ failure Chest 1992 101 6 1481 1483 10.1378/chest.101.6.1481 1600757

[B25] Angus DC Pereira CA Silva E Epidemiology of severe sepsis around the world Endocr Metab Immune Disord Drug Targets 2006 6 2 207 212 16787296 10.2174/187153006777442332

[B26] Marshall JC Measures, markers, and mediators: toward a staging system for clinical sepsis. A report of the Fifth Toronto Sepsis Roundtable, Toronto, Ontario, Canada, October 25-26, 2000 Crit Care Med 2003 31 5 1560 1567 10.1097/01.CCM.0000065186.67848.3A 12771633

[B27] Roch A NH2 terminal pro-brain natriuretic peptide plasma level as an early marker of prognosis and cardiac dysfunction in septic shock patients Crit Care Med 2005 33 5 1001 1007 10.1097/01.CCM.0000162561.82012.E9 15891328

[B28] Gibot S Time-course of sTREM (soluble triggering receptor expressed on myeloid cells)-1, procalcitonin, and C-reactive protein plasma concentrations during sepsis Crit Care Med 2005 33 4 792 796 10.1097/01.CCM.0000159089.16462.4A 15818107

[B29] Ewig S Welte T Biomarkers in the diagnosis of pneumonia in the critically ill: don't shoot the piano player Intensive Care Med 2008 34 6 981 984 10.1007/s00134-008-1088-6 18392806

[B30] Challen K Evaluation of triage methods used to select patients with suspected pandemic influenza for hospital admission Emerg Med J 2011 doi:10.1136/emj.2010.104380 10.1136/emj.2010.104380 21586758

[B31] Brandao-Neto RA The role of Pneumonia scores in the emergency room in patients infected by 2009 H1N1 infection Eur J Emerg Med 2011 doi: 10.1097/MEJ.0b013e328349ed85 10.1097/MEJ.0b013e328349ed85 21785358

[B32] Bohrer H Role of NFkappaB in the mortality of sepsis J Clin Invest 1997 100 5 972 985 10.1172/JCI119648 9276714 PMC508272

[B33] Gaini S High mobility group box-1 protein in patients with suspected community-acquired infections and sepsis: a prospective study Crit Care 2007 11 2 R32 10.1186/cc5715 17346334 PMC2206448

[B34] Lee N Hypercytokinemia and hyperactivation of phospho-p38 mitogen-activated protein kinase in severe human influenza A virus infection Clin Infect Dis 2007 45 6 723 731 10.1086/520981 17712756

[B35] de Jong MD Fatal outcome of human influenza A (H5N1) is associated with high viral load and hypercytokinemia Nat Med 2006 12 10 1203 1207 10.1038/nm1477 16964257 PMC4333202

[B36] Alleva LM Budd AC Clark IA Systemic release of high mobility group box 1 protein during severe murine influenza J Immunol 2008 181 2 1454 1459 18606700 10.4049/jimmunol.181.2.1454

[B37] Bopp C sRAGE is elevated in septic patients and associated with patients outcome J Surg Res 2008 147 1 79 83 10.1016/j.jss.2007.07.014 17981300

[B38] van Zoelen MA Achouiti A van der Poll T RAGE during infectious diseases Front Biosci (Schol Ed) 2011 3 1119 1132 10.2741/215 21622260

[B39] Yamagishi S Positive association between serum levels of advanced glycation end products and the soluble form of receptor for advanced glycation end products in nondiabetic subjects Metabolism 2006 55 9 1227 1231 10.1016/j.metabol.2006.05.007 16919543

